# Long-term prevalence of post-traumatic stress disorder symptoms in patients after secondary peritonitis

**DOI:** 10.1186/cc5710

**Published:** 2007-02-23

**Authors:** Kimberly R Boer, Cecilia W Mahler, Cagdas Unlu, Bas Lamme, Margreeth B Vroom, Mirjam A Sprangers, Dirk J Gouma, Johannes B Reitsma, Corianne A De Borgie, Marja A Boermeester

**Affiliations:** 1 Department of Clinical Epidemiology & Biostatistics, Academic Medical Center, Amsterdam, The Netherlands; 2 Department of Surgery, Academic Medical Center, Amsterdam, The Netherlands; 3 Department of Intensive Care Medicine, Academic Medical Center, Amsterdam, The Netherlands; 4 Department of Medical Psychology, Academic Medical Center, Amsterdam, The Netherlands

## Abstract

**Introduction:**

The aim of this study was to determine the long-term prevalence of post-traumatic stress disorder (PTSD) symptomology in patients following secondary peritonitis and to determine whether the prevalence of PTSD-related symptoms differed between patients admitted to the intensive care unit (ICU) and patients admitted only to the surgical ward.

**Method:**

A retrospective cohort of consecutive patients treated for secondary peritonitis was sent a postal survey containing a self-report questionnaire, namely the Post-traumatic Stress Syndrome 10-question inventory (PTSS-10). From a database of 278 patients undergoing surgery for secondary peritonitis between 1994 and 2000, 131 patients were long-term survivors (follow-up period at least four years) and were eligible for inclusion in our study, conducted at a tertiary referral hospital in Amsterdam, The Netherlands.

**Results:**

The response rate was 86%, yielding a cohort of 100 patients; 61% of these patients had been admitted to the ICU. PTSD-related symptoms were found in 24% (95% confidence interval 17% to 33%) of patients when a PTSS-10 score of 35 was chosen as the cutoff, whereas the prevalence of PTSD symptomology when borderline patients scoring 27 points or more were included was 38% (95% confidence interval 29% to 48%). In a multivariate analyses controlling for age, sex, Acute Physiology and Chronic Health Evaluation II (APACHE II) score, number of relaparotomies and length of hospital stay, the likelihood of ICU-admitted patients having PTSD symptomology was 4.3 times higher (95% confidence interval 1.11 to 16.5) than patients not admitted to the ICU, using a PTSS-10 score cutoff of 35 or greater. Older patients and males were less likely to report PTSD symptoms.

**Conclusion:**

Nearly a quarter of patients receiving surgical treatment for secondary peritonitis developed PTSD symptoms. Patients admitted to the ICU were at significantly greater risk for having PTSD symptoms after adjusting for baseline differences, in particular age.

## Introduction

Peritonitis or abdominal sepsis is a severe disease with high mortality (approximately 30%) [1,2]. Intensive care unit (ICU) and hospital admission may be lengthy and morbidity extensive. Hence, experiencing peritonitis is a major life event. Patients who survive critical illness often report poor quality of life and exhibit post-traumatic stress disorder (PTSD) symptomology during the post-clinical period [3-8]. PTSD symptoms include intrusive recollections, avoidant/numbing symptoms and hyperarousal symptoms resulting from exposure to one or more traumatic events [9]. Patients with PTSD (symptoms) have reduced quality of life [6,8,10-12] and frequently suffer from depression [8,13]. Therefore, monitoring PTSD symptomology in ICU patients could complement hospital and long-term survival outcomes, guide early sociopsychological interventions and improve long-term patient care. Hence, it is worth evaluating PTSD in order to elucidate the complex nature of long-term outcomes in this setting [14].

Many survivors of critical illness and its treatment suffer from continuous traumatic memories and re-live adverse experiences from their illness, such as respiratory distress, anxiety, pain and loss of control, which are all associated with an increased risk for development of PTSD [3,6]. Studies have reported prevalence rates of 15% to 38% for PTSD-related symptoms in patients who had been admitted to the ICU [4,8]. Some authors have argued that specific circumstances and memories during the ICU stay can serve as a trigger for developing PTSD symptoms rather than having a severe underlying illness itself. However, the majority of studies examining the relation between ICU stay and PTSD symptoms were conducted in cohorts in which all patients had been admitted to the ICU, rendering these studies unable to differentiate between ICU and non-ICU patient experiences.

In addition, data on the prevalence of PTSD-related symptoms following secondary peritonitis are lacking. It is unknown whether the prevalence of symptoms related to PTSD or memories of traumatic experiences differ between peritonitis patients after ICU admission (who have undergone surgery, ICU stay and hospital ward stay) and patients without ICU admission (who have undergone surgery and hospital ward stay only).

The aim of the present study was first to determine the long-term prevalence of PTSD symptomology in patients 4 to 10 years after secondary peritonitis based on a self-report questionnaire. We also aimed to compare the prevalence of PTSD-related symptoms between patients admitted to the ICU and patients admitted only to the surgical ward. Finally, we examined whether the prevalence of PTSD symptomology in these patients was increased because of the traumatic memories that patients had during their ICU and/or hospital stay [1].

## Materials and methods

### Study population

A retrospective cohort of 278 consecutive patients, who were treated surgically for secondary peritonitis between January 1994 and January 2000, was the starting cohort in the study [1]. All patients were treated at the Department of Surgery in the Academic Medical Center at the University of Amsterdam, The Netherlands. All patients who were still alive at follow up were eligible for inclusion. These patients were informed about the study by telephone in order to improve the response rate. Because of the noninterventional nature of the study, the institutional review board waived the need for informed consent.

### Data collection

All patients still alive at follow up were eligible for the study (*n *= 118) and received a standardized instrument for assessing symptoms related to PTSD, namely the Posttraumatic Stress Scale 10-question inventory (PTSS-10). In addition, they received a four-question Adverse Experiences Questionnaire. Each questionnaire addressed the patient's feelings over the preceding 14 days. Patients who had been admitted to the ICU during their hospital stay for peritonitis were sent a questionnaire that specifically asked the patient to consider their feelings during the preceding 14 days while keeping their past ICU stay in mind. Patients not admitted to the ICU were asked to complete the questionnaire for the preceding 14 days keeping in mind their past stay in the general ward following their episode of peritonitis.

A separate questionnaire was included to collect relevant clinical data following discharge from the hospital for peritonitis (including readmissions since discharge after surgical treatment for secondary peritonitis and use of medication during the preceding few years, and newly developed diseases and their treatment).

Patients who returned incomplete questionnaires were contacted by phone within two weeks in an attempt to complete the questionnaire by phone. Patients who did not return the questionnaires were sent the questionnaires two more times within a six week period. After these attempts had been made, patients who had given initial telephone consent were contacted again to obtain information regarding their motivations for not responding.

Demographic and clinical data at the time of the index surgical procedure (the emergency laparotomy performed at initial presentation of peritonitis) were collected from hospital charts and computerized registration system. The following information was recorded: age, sex, comorbidity, use of medication, Acute Physiology and Chronic Health Evaluation [APACHE] II score before surgery and Mannheim Peritonitis Index (MPI). Disease and surgical characteristics recorded contained aetiology of peritonitis, origin of peritonitis, surgical treatment strategy and number of relaparotomies. Postoperative characteristics recorded included the number of days spent in hospital, the number of days spent in the ICU, days of mechanical ventilation, 'open abdomen' (laparostomy) during admission, number and type of complications, number of readmissions and the mean follow-up time. Patient recall was checked using the hospital information and medication system to check readmission and use of medication. Details regarding out-of-hospital medications, such as those prescribed by the family physician, were obtained only by questionnaire.

### Instruments

#### Post-traumatic Stress Syndrome 10-question inventory

The PTSS-10 was originally designed to diagnose PTSD, according to Diagnosis and Statistical Manual of Mental Disorders (DSM)-II criteria, in victims of natural disasters [15], and it was subsequently validated in Norwegian seaman after they had undergone torture in Libya [16]. The PTSS-10 has since been validated in patients with acute respiratory distress disorder (ARDS) after ICU treatment using the Structured Clinical Interview for DSM-IV (SCID) Axis II Personality Disorders [9]. The PTSS-10 is now a widely used and validated self-report questionnaire; it has been reported to achieve a sensitivity of 77% and a specificity of 97.5% for the diagnosis of PTSD [17].

The questionnaire consists of 10 items, each with a Likert scale ranging from 1 ('never') to 7 ('always'). A summated score with a range between 10 and 70 is calculated, with higher scores indicating more PTSD-related symptoms. A score of 35 or greater is considered an adequate cutoff for PTSD-related symptomology [11,17-19], whereas patients with scores between 27 and 35 on PTSS-10 were considered to have borderline PTSD symptomology. The validated English version was translated into Dutch according to a forward-backward translation procedure.

#### Adverse events/traumatic experiences questionnaire

The four-item Adverse Experiences Questionnaire assesses the presence of four types of traumatic memories during a stay in the ICU or hospital ward [17]: anxiety, respiratory distress, pain, and/or nightmares. Patients scored the frequency with which they experienced these traumatic events (or their recollection of them) during their stay in the ICU or hospital ward using a 4-point response scale: 1 = none, 2 = sometimes, 3 = regularly and 4 = often.

### Analysis

Ninety-five per cent confidence intervals around estimates of prevalence were calculated using the method of Wilson [20]. Clinical characteristics and the prevalence of PTSD symptoms between patients who were admitted to ICU during their initial stay and those who were treated solely on the surgical ward were compared. Depending on the nature of the clinical variables, we used Pearson χ^2^, Student's *t*, or Mann-Whitney *U *tests.

We built multivariate logistic regression models to assess the association between ICU stay and the presence of PTSD symptomology (PTSS-10 score >35) after adjusting for other factors. We adjusted for factors related to patient characteristics (age [continuous] and sex), disease characteristics (APACHE-II score at baseline [continuous] and whether patients had undergone one or more relaparotomies [yes/no]) and postoperative characteristics (days spent in hospital [transformed to base 10 in order to improve the linear relationship with outcome]) [21]. These factors were chosen either because they were identified in earlier PTSD studies and literature [21] (for instance, age, sex and comorbidity) or because they exhibited univariate significance (*P *< 0.1) with the dependent factor (PTSD symptomology) in our study (APACHE II score, patients undergoing more than one relaparotomy and days in hospital). If factors were highly correlated, we selected only one of the correlated factors in the multivariate model to avoid the problem of co-linearity. Odds ratios with 95% confidence intervals (CIs) were used to quantify the strength of the association. To determine the fit of the final multivariate logistic model, we calculated the area under the receiver operating characteristic curve, also known as the concordance statistic, and performed the Hosmer-Lemeshow goodness-of-fit test.

To determine whether traumatic memories acquired during the stay in hospital or the ICU played a role in the development of PTSD symptomology, we examined the percentage of patients with PTSD symptomology within each level of response on the traumatic memories questions. Because of the ordered response on the traumatic memories questions, we used the χ^2 ^test for trend to examine this relation.

*P *< 0.05 were considered statistically significant.

## Results

**Figure 1:**
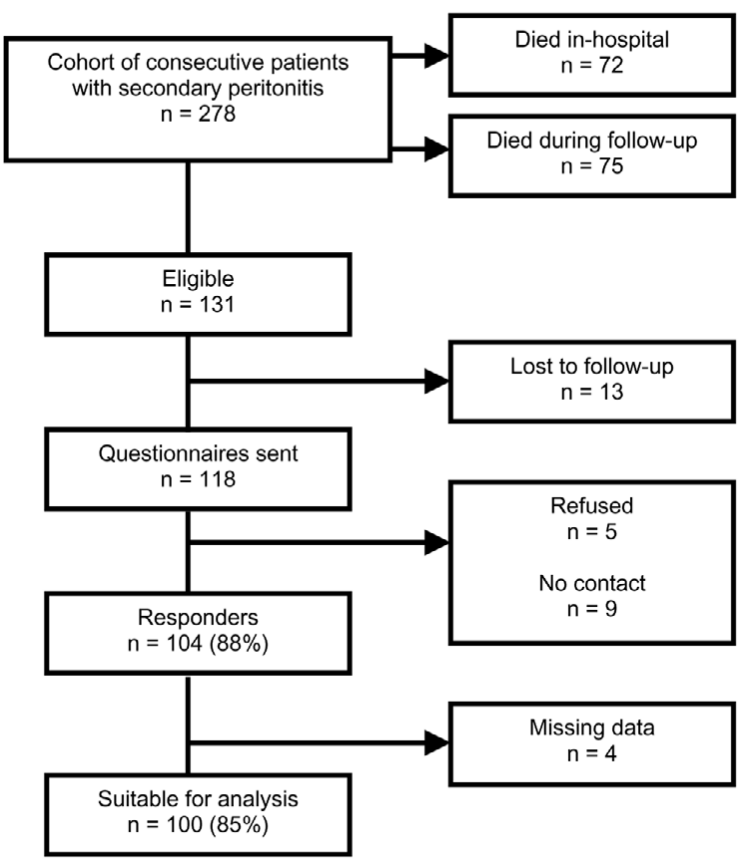
Flowchart of study inclusion.

From the initial cohort of 278 patients with secondary peritonitis [1], 118 patients were long-term survivors. These patients received the set of questionnaires, and 104 patients (88%) responded (Figure 1). Of the 14 patients who did not respond to the questionnaire, five patients were not willing to complete the questionnaire and nine patients, who were initially informed about the study by phone before the mailing, could not be contacted again to find out the reason for not responding to the questionnaire. Four patients were excluded because too many data were missing (Figure 1). No significant differences in operative, hospital-related, or postoperative characteristics were found between patients suitable for analysis (*n *= 100) and eligible patients still alive who did not respond (*n *= 32). However, comparison of patient characteristics between the two groups revealed that patients in the nonresponding group were younger (mean 51 years versus 40 years; *P *< 0.001), presented with fewer comorbidities (comorbidity present in 65% versus 30%; *P *< 0.001) at initial surgery, and had lower APACHE II scores (9.5 versus 7.5; *P *= 0.049) and MPI scores (22 versus 18.6; *P *= 0.024) than did patients in the responder group. There was no difference between responding patients and nonresponders in ICU admittance.

In responding patients the average interval between index operation and follow up was 5.3 years for ICU and non-ICU patients. Comorbidity was present in 65% of patients, and nearly 80% of patients were on some type of medication (Table 1). The APACHE II score (mean ± standard deviation) at the time of the index operation was 9.5 ± 5 and the MPI score was 21.9 ± 7. Seventy-six per cent of patients were treated using an on-demand relaparotomy strategy and 24% were managed according to a planned relaparotomy strategy; overall, 59% of patients underwent one or more re-laparotomies.

**Table 1:** Characteristics of study population

Type of characteristics	Patients			*P *value (non-ICU versus ICU)
	Overall (*n *= 100)	Non-ICU (*n *= 39)	ICU (*n *= 61)	

Patient characteristics at index operation				

Age (years; mean ± SD)	51.1 ± 14	46.7 ± 15	54.2 ± 13	0.011^a^
Male sex (% [*n*])	59% (60)	51% (20)	64% (39)	0.21^b^
Comorbidity (% [*n*])	65% (66)	67% (26)	65% (40)	0.91^b^
Use of any medication (% [*n*])	77% (78)	82% (32)	78% (46)	0.62^b^
APACHE II score (mean ± SD)	9.5 ± 5.2	8.2 ± 5	10.4 ± 5	0.037
MPI score (mean ± SD)	21.9 ± 7.4	20.0 ± 8	23.2 ± 7	0.036^a^

Disease and operative characteristics				

Aetiology of peritonitis (% [*n*])				0.29^b^
Perforation	39% (39)	42% (17)	36% (22)	
Anastomotic leakage	26% (26)	25% (10)	26% (16)	
Ischaemia	5% (5)	-	8% (5)	
Pancreatitis	7% (7)	2% (1)	9% (6)	
Bile leakage	7% (7)	10% (4)	4% (3)	
Abcess	9% (9)	7% (3)	9% (6)	
Other	7% (7)	12% (4)	4% (3)	
Origin of peritonitis (% [*n*])				0.28^b^
Colon	36% (36)	38% (15)	34% (21)	
Small intestine	25% (25)	25% (10)	25% (15)	
Pancreas	13% (13)	3% (1)	20% (12)	
Appendix	4% (4)	5% (2)	3% (2)	
Gall bladder	4% (4)	5% (2)	3% (2)	
Stomach/duodenum	6% (6)	5% (2)	7% (4)	
Other	12% (12)	21% (7)	8% (5)	
Treatment strategy (% [*n*])				0.11^b^
On demand	76% (77)	85% (33)	71% (43)	
Planned relaparotomy	24% (24)	15% (6)	29% (18)	
Laparostomy (open abdomen) during admission (% [*n*])	25% (25)	8% (3)	36% (22)	0.001^b^
Patients with = 1 relaparotomy (% [*n*])	59% (60)	40% (16)	72% (44)	0.002^b^

Postoperative characteristics				

Days in hospital (median [25th to 75th percentile])	37.0 (21 to 55)	27.0 (17 to 41)	49.0 (27 to 73)	< 0.001^a^
Days in ICU (median [25th to 75th percentile])^c^	-	-	16.0 (5 to 30)	NA
Patients mechanically ventilated (% [*n*])^c^	-	-	89% (54)	n.a.
Days of mechanical ventilation (median [25th to 75th percentile])^c^	-	-	11.0 (4 to 25)	n.a.
Complications (% [*n*])				
Surgery related	63% (67)	58% (23)	72% (44)	0.17^b^
Sepsis related	38% (38)	8% (3)	57% (35)	< 0.001^b^
Readmission to hospital (% [*n*])	14% (14)	18%(7)	12% (7)	0.37^a^
Time since index operation				
Time of questionnaire receipt since index operation (months; median [min-max])	88.6 (49 to 127)	88.4 (50 to 122)	88.5 (49 to 127)	0.99^a^

^a^T-test or Mann-Whitney *U*-test. ^b^Pearson's or Fischer's exact χ^2^. ^c^Only patients who were admitted to the ICU (*n *= 61). APACHE, Acute Physiology and Chronic Health Evaluation; ICU, intensive care unit; MPI, Mannheim Peritonitis Scale; NA, not applicable; SD, standard deviation.

### Post-traumatic stress disorder symptomology

The median PTSS-10 score among all patients was 22, with 25% of the patients having a score below 13 and 25% of patients with a score above 33. Using the recommended cutoff value for PTSD symptomology of 35 points on the PTSS-10 questionnaire [17,19,22,23], the overall prevalence of PTSD-related symptoms was 24% (95% CI 17% to 33%). The overall prevalence of PTSD symptomology including borderline patients who scored 27 points or more was 38% (95% CI 29% to 48%).

### Comparison between ICU and non-ICU patients

Patient, disease and operative characteristics for ICU patients (61%) and non-ICU patients (39%) are presented in Table 1. Patients who had had an ICU stay were on average 7.5 years older than patients who were not admitted to the ICU (*P *= 0.011). ICU patients also had higher APACHE II score (mean difference 2.2 points; *P *= 0.037) and MPI score (mean difference 3.2 points; *P *= 0.036). Of patients who had had an ICU stay 36% underwent laparostomy (open abdomen), whereas only 8% of the ward patients underwent laparostomy (in 92% of patients admitted to the surgical ward primary abdominal closure was done; *P *= 0.001). A relaparotomy was significantly more common in the ICU group than in the non-ICU group (72% versus 40%; *P *< 0.001).

With respect to postoperative characteristics, patients had a median stay in hospital of 37 days. ICU survivors had a longer hospital stay than did non-ICU survivors (median days: 49 versus 27; *P *= 0.001) and suffered more nonsurgical complications (57% versus 8%; *P *< 0.001). Fifty-four (89%) patients required mechanical ventilation during their ICU stay. These patients were ventilated for a median of 11 days. Four of the ICU-admitted patients suffered early ARDS (within 96 hours) following peritonitis.

### Post-traumatic stress disorder symptoms

In an univariate analysis, using a PTSS-10 score above 35 as the cutoff, we found a prevalence of PTSD symptomology of 18% (7/39) in the non-ICU group and 28% (17/61) in the ICU group (*P *= 0.21). We examined several factors to determine whether they confounded the strength of the relationship between ICU stay and the probability of having relevant PTSD symptomology (Table 2). After controlling for age, sex, APACHE II score, relaparotomy and length of hospital stay in a multivariate analysis, patients admitted to the ICU were more likely to report PTSD symptomology on the PTSS-10 questionnaire than patients admitted to the surgery ward only (odds ratio [OR] 4.3, 95% CI 1.11 to 16.5).

**Table 2:** Multivariable logistic regression analysis for factors associated with the presence of PTSD symptomology

	PTSS-10 sum score		Adjusted OR^a ^(95% CI)
		
	Patients with scores > 35 (*n *= 24)	Patients with scores < 35 (*n *= 76)	
Patients admitted to the ICU (%)	71%	57%	4.3 (1.11 to 16.5)
Females (%)	54%	37%	3.5 (1.2 to 10.6)
Age (years; mean ± SD)	52.9 ± 14.9	59.8 ± 14.1	0.93^b ^(0.89 to 0.98)
APACHE II score (mean ± SD)	10.7 ± 5.8	9.1 ± 5.0	1.1^c ^(1.002 to 1.25)
≥ 1 Relaparotomy (%)	63%	59%	3.8 (0.86 to 16.8)
Hospital stay (days; median [25th to 75th percentile])^d^	46 (28 to 54)	33 (21 to 59)	2.2^e ^(0.8 to 5.8)

^a^The odds ratio (OR) has been adjusted for sex, age, Acute Physiology and Chronic Health Evaluation (APACHE) II score, ≥ 1 relaparotomy and length of hospital stay. ^b^Per one-year increase in age, the odds ratio (OR) for having post-traumatic stress disorder (PTSD) symptoms decreased by 0.93. ^c^Per one-point increase in APACHE II score, the OR for having PTSD symptoms increased by 1.1. ^d^In the logistical model hospital stay is log to the base 10 transformed. ^e^For one patient hospital discharge dates were missing, and therefore information regarding length of hospital stay was missing; imputation was done using the mean duration of stay for the non-ICU stay group; one patient was missing APACHE II score data. PTSS-10, Post-traumatic Stress Syndrome 10-question inventory; SD, standard deviation.

Other factors that were significantly associated with more PTSD symptoms in the multivariate model included gender, age, and severity of disease at initial surgery. Females were more likely to develop PTSD symptoms than were males (OR 3.5, 95% CI 1.2 to 10.6). With every one-year decrease in age, the likelihood of developing PTSD symptoms decreased (OR 0.93, 95% CI 0.89 to 0.98). Finally, with every point increase in APACHE-II score, the chances of developing PTSD symptoms increased (OR 1.1, 95% CI 1.002 to 1.25). Therefore, the main reason for finding a stronger relation between ICU stay and PTSD symptomology in the multivariate model is that older patients are less likely to develop PTSD symptoms. Because ICU patients on average were older than non-ICU patents, the unadjusted relationship underestimated the effect of ICU on PTSD symptoms. Males were also less likely to report PTSD symptomology (OR 0.95, 95% CI 0.91 to 0.98), but because of the comparable sex distribution in ICU and non-ICU patients it did not confound the relation between ICU stay and PTSD symptomology (Table 2). Length of hospital stay was associated with more PTSD symptomology, and it was therefore also a confounder for the relation between ICU stay and PTSD symptomology because hospital stay was markedly longer in ICU patients than in non-ICU patients. The area under the receiver operating characteristic curve for the final multivariate model was 0.77 (95% CI 0.66 to 0.88). This indicates that if we were to randomly choose one patient above the PTSS-10 cutoff value and one patient below, the probability that the patient above the cutoff would have a higher predicted risk for PTSD symptomology based on the model is 79%. Differences in observed versus predicted probabilities were small, with the Hosmer-Lemeshow test yielding a *P *value of 0.41.

### Traumatic memories and symptoms of post-traumatic stress disorder

In the total study population, traumatic memories were associated with more PTSD symptomology (Table 3). Patients reporting more traumatic memories during their ICU or hospital stay reported significantly more PTSD symptoms on the PTSS-10. Patients with nightmares, panic attacks, intense pain and difficulty breathing during their ICU or hospital ward stay had higher median scores than did patients reporting no traumatic memories from the ICU or hospital ward (Table 3). There were, however, no statistically significant differences between the ICU group and the non-ICU group of patients with respect to reporting of traumatic memories (nightmares: χ^2 ^= 5.84, *P *= 0.12; fear or panic attacks: χ^2 ^= 6.9, *P *= 0.075; pain: χ^2 ^= 1.01, *P *= 0.80; and difficulty breathing: χ^2 ^= 5.3, *P *= 0.15).

**Table 3:** Traumatic memories during ICU/hospital stay in relation to PTSS-10 score

Traumatic memories or adverse experiences during ICU/hospital stay	Percentage of patients with PTSS-10 sum score above 35 (*n *= 24)	*P *value (for trend)^a^
Nightmares		
Never (*n *= 42)	9.5	
Sometimes (*n *= 29)	24.1	0.001
Regularly (*n *= 20)	45.0	
Often (*n *= 9)	44.4	
Fear or panic attacks		
Never (*n *= 53)	9.4	
Sometimes (*n *= 23)	26.1	< 0.001
Regularly (*n *= 16)	50.0	
Often (*n *= 8)	62.5	
Intense pain		
Never (*n *= 27)	7.4	
Sometimes (*n *= 35)	17.1	0.007
Regularly (*n *= 15)	60.0	
Often (*n *= 23)	30.4	
Difficulty breathing		
Never (*n *= 50)	12.0	
Sometimes (*n *= 30)	33.3	0.014
Regularly (*n *= 9)	44.4	
Often (*n *= 11)	36.4	

^a^χ^2 ^test for linear trend. ICU, intensive care unit; PTSS-10, Post-traumatic Stress Syndrome 10-question inventory.

## Discussion

Our cohort of patients experiencing the same acute disease includes both patients who have been admitted to the ICU and those who were treated on the surgical ward only. This enabled us to conduct a detailed analysis of the impact of ICU stay on long-term PTSD symptomology. We found a high overall prevalence of long-term PTSD symptomology, as indicated by the PTSS-10 questionnaire, many years after surgical treatment for secondary peritonitis. The proportion of patients scoring above the 35-point threshold on PTSS-10 was 24%.

The PTSS-10 is an instrument specifically designed to identify PTSD symptoms in ICU patients. The prevalence of PTSD symptoms in our patients was similar to that in a retrospective study conducted in ARDS patients in 1998 using the PTSS-10 [6], and it was similar to that in ARDS patients studied in 2004 (median follow up eight years) in which 24% of patients suffered full-blown PTSD (as diagnosed using SCID) [11]. Past studies found a lifetime prevalence of 7.8% to 8.3% in the US general population in the 1990s [24], but more recently a study conducted in six European countries (the European Study Of The Epidemiology Of Mental Disorders [ESEMeD] study) [25,26] estimated a considerably lower prevalence of PTSD, varying between 0.9% and 2.9%. Compared with these general populations, the proportions of patients from an ICU population with PTSD symptomology, a considerable time after discharge, are high [13,25-28].

We found that patients who responded to the PTSS-10 questionnaire exhibited higher APACHE II scores and MPI scores, and increased comorbidity than did patients who did not respond to the questionnaire. These differences might have led to a small overestimation of the prevalence of PTSD symptoms (*n *= 100). However, our patient group had an overall lower mean APACHE II score than that reported in other ICU populations with similar prevalence of PTSD symptoms. Although the APACHE II scores of patients admitted to the ICU in our study are lower than those in other studies on PTSD symptoms using the PTSS-10 questionnaire [3,4], the APACHE II scores are not particularly low for a population of patients with peritonitis [1].

In a univariate analysis we found no significant differences in the prevalence of PTSD symptoms between ICU (28%) and non-ICU patients (18%) on the PTSS-10. However, ICU stay was independently associated with PTSD symptomology after adjusting for other factors related to PTSD, in particular age. As expected, when comparing ICU patients with non-ICU patients, differences were found in patient, disease, operative and postoperative characteristics. ICU patients were older and had more severe disease (based on the recorded APACHE II score), more surgical interventions and longer hospital stay, all of which could have affected their eventual PTSD symptomology. To control for these differences and to determine whether ICU was an independent factor for PTSD, we created a multivariate model. When controlling for age, sex, APACHE II score, having undergone one or more relaparotomy, and length of hospital stay in the postoperative period, we found a significant difference in the prevalence of PTSD symptomology (based on PTSS-10 score) between patients with and without an ICU stay. Older age and being male had a protective role, whereas higher APACHE II scores led to more PTSD symptoms. These findings are in contrast to earlier data, in which no associations between higher APACHE II score and greater probability of developing of PTSD symptoms were identified [4,5]. It is important to note that even the non-ICU group exhibited a relatively high prevalence of PTSD-related symptoms. This suggests not only the ICU environment but also secondary peritonitis *per se *may be a sufficiently traumatic event for a patient to develop PTSD.

Because mechanical ventilation has previously been associated with development of more PTSD-like symptoms after ICU treatment [29], this might be the reason why our ICU patients also exhibited more PTSD symptomology than did the surgery ward only patients. Because nearly all of our ICU patients were mechanically ventilated, we could not determine the independent impact of these two factors.

Because of the retrospective nature of the study, details concerning the severity of sepsis (such as septic shock status on admission and hydrocortisone use during the ICU stay) could not be ascertained as risk factors in all patients [12,23]. These factors could be important in the development of PTSD symptoms in ICU patients. The importance of hydrocortisone use in the ICU and the development of PTSD symptoms has previously been highlighted [12,23]. A randomized study [23] showed that introduction of hydrocortisone treatment into the regimen during an ICU stay reduces subsequent development of PTSD symptoms. In past studies ARDS has been demonstrated to be an independent predictor of developing PTSD symptoms; patients suffering from ARDS were found to exhibit more PTSD symptoms [6,10,11], but in the present study we only had data on development of ARDS within the first four days after peritonitis. Risk factors in the ICU environment such as ARDS, septic shock and mechanical ventilation (the vast majority of the study patients admitted to the ICU were ventilated) could, at least in part, account for the differences in PTSD symptoms between patients in the ICU those those managed on surgical wards only.

There were no differences in the number of traumatic memories reported between ICU patients and the patients managed on the surgical ward only, although we found a clear positive linear association between more traumatic memories and higher scores on the PTSS-10. This relation between traumatic memories and the PTSS-10 score was also found in two earlier studies conducted in ICU patients [6,30]. We anticipated that the ICU environment would create more traumatic memories, which would in turn lead to more PTSD-related symptoms. However, the proportion of patients with traumatic memories was comparable between ICU and non-ICU patients.

### Limitations

Ideally, PTSD is diagnosed using a SCID [23], in accordance with the DSM-IV [9]. SCID is a semi-structured diagnostic interview designed to allow clinicians and researchers to make reliable DSM-IV psychiatric diagnoses. In recent studies it has been established that a self-report PTSS-10 questionnaire can be as useful a tool in determining which patients are suffering from PTSD symptomology [5,17]. These studies found significantly higher PTSS-10 scores in patients with a SCID-II PTSD diagnosis than in patients without. The sensitivity in these studies varied from 77% to 100%, and specificity from 92% to 98% when using a cutoff score of 35 [5,17]. However, these estimates were imprecise because of the small sample sizes in these studies. It is unclear to what extent sensitivity and specificity of the PTSS-10 instrument for PTSD may vary according to disease and other characteristics [31].

Recognition of the distinction between PTSD symptoms captured by the PTSS-10 and a PTSD diagnosis is vital, because this questionnaire does not give a DSM-IV diagnosis but only an indication of the level of PTSD symptomology. Clinically, a score on the PTSS-10 above the cutoff should prompt the attending physician to refer the patient to a psychologist to conduct a SCID [9].

Our results suggest that the (persisting) presence of traumatic memories is likely to be relevant to development of PTSD-related symptoms following a traumatic event, and not the ICU stay alone, because we observed a strong linear relationship between traumatic memories and PTSS-10 score. We assessed these traumatic memories (or adverse experiences) in accordance with patients' recollections. This may limit the conclusions one can make, because it is possible that perception of a traumatic experience may contribute to long-term PTSD symptomology, hence making a causal conclusion impossible.

Information concerning other unrelated traumatic experiences or life events that may have occurred after hospital admission was not collected. Therefore, the influence of superimposed trauma cannot be ruled out [6]. Also, because this was a retrospective study, it was also not possible to collect PTSD data on patients before their peritonitis. However, considering the acute nature of peritonitis, it would be difficult to collect such data even in a prospective trial. Given the impact of a severe life-threatening illness such as peritonitis, a relationship with the development of PTSD symptoms is plausible, but causality cannot be established when no information is available on other life events.

## Conclusion

Nearly a quarter of patients receiving surgical treatment for secondary peritonitis developed PTSD symptoms. Considering the high long-term prevalence of PTSD, patients admitted to the ICU had a higher risk for PTSD symptoms but only after taking their higher age into account. Early detection of PTSD in peritonitis patients by questionnaires such as the PTSS-10 deserves attention.

## Key messages

• In a cohort of 100 patients with secondary peritonitis, of whom 61 were admitted to the ICU and 39 were not (admitted to the surgical ward only), the overall prevalence of long-term PTSD symptomology using the PTSS-10 questionnaire was 24%.

• In a univariate analysis we found no differences in PTSS-10 scores between ICU and non-ICU patients, but ICU stay was significantly associated with PTSD symptomology after adjusting for other factors related to PTSD, in particular age.

• There were no differences in the number of traumatic memories reported between ICU patients and patients managed on the surgical ward only, although we found a clear positive linear association between more traumatic memories and higher scores on the PTSS-10.

## Abbreviations

APACHE = Acute Physiology and Chronic Health Evaluation; ARDS = acute respiratory distress syndrome; CI = confidence interval; DSM = Diagnostic and Statistical Manual of Mental Disorders; ICU = intensive care unit; MPI = Mannheim Peritonitis Scale; OR = odds ratio; PTSD = post-traumatic stress disorder; PTSS-10 = Post-traumatic Stress Syndrome 10-question inventory; SCID = Structured Clinical Interview for DSM-IV Axis II Personality Disorders.

## Competing interests

The authors declare that they have no competing interests.

## Authors' contributions

MB, DG, MV and BL designed the study and advised on surgical and ICU information; all information pertaining to surgical procedures and ICU stay for the final manuscript were considered by MB and BL. CM, BL and CU were responsible for the coordination of the study. CU and CM contacted patients, and collected and entered data. MS and CB advised for all quality of life and PTSD issues. KB, HR and MB analyzed data, and KB was responsible for the final manuscript. KB, CB, HR, MS and MB interpreted and discussed all data. All authors read and approved the final manuscript.
